# Rambling Notes and Reflections, Suggested during a Visit to Paris in the Winter of 1826-7

**Published:** 1828-01-01

**Authors:** 


*Rambling Notes and Reflections, suggested during a Visit to Paris in the Winter of* 1826—1827. By Sir ARTHUR BROOKE FAULKNER. Octavo, pp. 348. London, August, 1827.

SOME time ago, we introduced to our readers the amusing rambles of Dr. Valentine, and regretted that he had not kept more closely to medical matters, leaving politics, general science, literature, and antiquities, to others who made those subjects their special study. Doubtless it is difficult for medical men of high classical education and general scientific attainments, to resist the temptation to remark on all subjects that come in their way, when journeying in foreign countries; and, as there is no rule against such indulgences, we cannot quarrel with either Dr. Valentine or Sir Arthur Faulkner for diverging into so many discussions totally unconnected with medicine. Neither can they find fault with us if, adhering to the precept "ne sutor ultra crepidam," we only take notice of those portions of their works which treat of professional subjects.

We are rejoiced to find so much religion among our provincial, and especially our fashionable physicians. Sir Arthur has occupied a considerable portion of his book with matters of the church, and wittily hopes, that this digression "will be considered, at least, a *clergyable* offence." As this is not a tribunal for such causes, we pass on to medical concerns. Sir Arthur's remarks are chiefly satirical, and levelled, of course, at the foibles, the intrigues, the arts, and, lastly, the delinquencies, of the disciples of Esculapius—including the various species and gradations, from the President of the College of Physicians down to "the Apothecaries' deputy." Satire, no doubt, has its advantage, when levelled against folly and knavery; but, if directed pretty generally against a whole profession, and especially the medical profession, we may well doubt whether considerable injury may not be done to the innocent, by prejudicing the non-professional part of the public. Satire of this kind too, is the more dangerous when dispensed in a work designed for general perusal. That the vices, the follies, and the knavery described by Sir Arthur, are to be found in the ranks of medical society, no one can deny; but the question is, are they so often and so abundantly found as to authorise the lash of satire being laid generally on the professional back? With every deference and respect for the learning, talents, and honorable character of Sir A. Faulkner, we find ourselves obliged to combat some of the inferences which he draws, and repel, or, at least, *blunt* some of the arrows of satire so plentifully discharged among the ranks of his brethren. Thus, comparing law with divinity and physic, Sir A. observes:—"The learned department of the Law differs from its sister-professions in one most paramount and essential respect; namely, that there is a direct and indissoluble connexion between *ability* and *success*; while, in the others, absolutely no more than between the value of the parsonage and the piety of its owner—the length of the doctor's bill and the modesty that drew it up." This evidently and literally means that there is no sort of connexion whatever between ability and success in the medical profession! Is Sir A. justified in this assertion? Have the many men who figured at the head of our profession during the last 30 or 40 years, owed nothing of their success and reputation to ability? Or was it all mere chance—nay, was it all owing to artful manœuvering? One of these must be the case, if there be no possible connexion between ability and success. That a crafty, unprincipled, but ignorant, medical man may occasionally rise to a *certain extent* of success and riches, we will not deny—and this, by the way, is the case in law as well as in physic; but, that such a character can ever attain or maintain any of the higher grades of emolument or reputation, we venture to deny. We appeal to the *living race* of medical men in this and other countries, for unequivocal proof of this position :—and if our position be correct, what becomes of the sweeping principle laid down by Sir Arthur? If, indeed, he had said, that there is no very necessary connexion between *learning* and *success*, he would have had some solid basis for his observation. There are numerous proofs among the living, as well as among the dead, that a man may be crammed brim full of literature and science, and yet he may not have the power of applying these acquisitions so as to obtain success. How often is the finest seed sown on a soil that never can render it prolific? Sir Arthur appears to us to have confounded *education* with *talent*—and to have thought (as will presently appear) that Oxford or Cambridge *should* "accomplish all things without grace"—that is, without native talent—but, in this, he is mistaken. An extensive and liberal education, wherever acquired, will undoubtedly give a man, in any of the three professions, a better chance of *success;* but it does not—and wisely is it ordained that it *should not, command* success, over superior native genius, which may happen to be destitute of the means of procuring this education through a particular and expensive channel. Suppose there was an ordonnance that no man should practise as a physician unless he first expended five thousand pounds on a University education. In this case, is it not evident, that *money* and *success* would almost be synonymous terms? We should think that money has quite enough of power and influence in its train, without making it the exclusive depository of knowledge also! To us, indeed, it appears, that one of the most potent antagonists to aristocratic wealth will be found in the popular diffusion of knowledge. That this sentiment is *secretly* shared by the aristocracy themselves, is quite evident from the anathemas poured by them on the New London University. We shall presently see that Sir Arthur is horror-stricken at the contemplation of this cheap mart of literature and science, divested of religious creeds, though one of his main principles, laid down in the very same work, is, "that *education* is the only security upon which any reliance can be placed that this most useful of all arts (the medical) is not abused to the vilest purposes, and converted into a curse instead of a blessing." *p.* 154. Sir Arthur is, to do him justice, a strenuous and eloquent advocate for education among all classes of society; but he is, like many others, terrified, because religion and religious creeds are not introduced into the new university. "How abominably horrible to contemplate being left to the mercy of a man's *philosophy*, as the only check upon his passions; who may as well take it into his head to think *cutting my throat as sound a piece of ethics as cutting his own.*" We would ask Sir Arthur if he ever met with any of these cut-throat ethics in his perusal of the ancient philosophers, who knew nothing of Christianity? His answer must be, no. Then why should philosophy *now* begin to teach the cutting of throats? Is Sir Arthur *serious* in preaching to us that it is the *religious* instruction imbibed at Oxford and Cambridge which makes the graduates of those universities so distinguished for their liberal conduct and moral ethics? We should think that he is laughing in his sleeve all the time that he is applying the flattering unction to the souls of the pious Christian and proud Aristocrat.

But we must leave generalities, and come to the professional portraits which our able, but too satirical, author has drawn. It has struck us, and we imagine it will also strike our readers, when perusing these portraits, that Sir Arthur either meant them to be complete caricatures—or that there is something in the air, the soil, or perhaps in the waters of Cheltenham, that has there congregated together a collection of quacks and demireps, such as no other part of this island could possibly produce. In either case, the propriety of handing up these pictures to society at large, who will not be slow in applying them generally to the profession, is at least very questionable. After giving some account of a medical character introduced on one of the Parisian stages, under the name of "*Le Medecin des Dames*," Sir Arthur proceeds as follows:—

"Alas! we need not travel far to find a match for this gentleman in our own honest land. It is humiliating to a profession, which deserves to be respectable, to name it, but I literally remember an M.D. in a good deal of business, fraught with one of the (ci-devant) 'Scotch licenses to slay,' who used to pay a certain number of hebdomadal visits, to perform the express service of catering gossip and mending the pens of a female patient; and she was amazingly taken with him. But why should we be surprised at this or any thing else of the kind, when we see the profession so very frequently in the hands of the ignorant; and that any man who chooses to practise *en docteur*, gets credit for skill only because he has stood a certain number of years behind a counter, or trod the wards of an hospital? The doctor, when speaking of his triplicate function, styles himself a general practitioner; and the general's course, I believe to be, too generally as follows. His first matriculation commences at the Galen's head, with little better preparation than a grocer's apprentice; and there he is doomed and indentured to remain for a certain number of years, pulverizing and extracting. Now, whatever he may claim for *extraction*, it will surely not be contended that such a place is just the most retired for *abstraction*. At least his snatches of opportunity for study, stolen from officinal hours and duties, cannot be very numerous; and allowing that he is ever so eager for scaling the heights of science, who is to direct his studies? His master must, like all other men, be liable to consult his own interest in preference to his apprentice's accomplishments, and to look for some more substantial return for his services, than the furniture of his brain, which is of so much less use to him than the produce of his hands: or, allowing that brain could not be entirely dispensed with, the work of his hands at least 'brings most grist to the mill.' As the indentures wear older, the apprentice begins to catch a little of the *auri sacra fames* himself, and seeing its gratification may be reached without the sciences, wisely abandons all farther thoughts of them, looking to more direct and available means. The indentures are now actually out, and the mature apprentice commences an established pharmacopolist on his own account. His course is now clear and straight before him. He pounds away through some profitable years, until he realizes a capital, and puts forth the bloom of his reputation, when, if the extent of his connexion gives him sufficient encouragement, (it will depend upon this,) he sloughs off his crysalis of gallipots, and expands into the many-coloured glories of the general practitioner. You then see him bustling (more frequently driving) from fistula to fever, until he comes to be looked upon as the very incarnate personification of the infallible pill he prescribes. But, without either colouring or exaggeration, there certainly is no profession within the whole range of respectable means of making a livelihood, the practice of which is so liable to deteriorate as physic, or one where a man, with a small smattering of knowledge, and a discreet cunning, may fleece with a safer freedom, or a more becoming grace, not only without risk of being detected, but even with character,—perhaps a high place in human esteem. And as we are on this subject, I shall trespass with my reasons for holding such an opinion. I begin, then, by assuming, that physic, *if* a trade, (the whole of my observations are hypothetical,) is *the* trade of all others the most exactly cut out for a rogue." 133.

"It is the fashion to talk of the daring impositions and profits of an imported mountebank: but I maintain that a homebred skark of our own, carries off more of the unrighteous mammon in a week, than your starveling of Italy in months. Give me a thorough-paced low grade of general practitioner, with a good *audacia perdita* and *sermo promptus*, and only one season or two of an hospital, I ask no more, I will back him in fame and profits against any dozen mere quacksalvers, and give you your choice of all Italy, from the Jura Alps to Calabria. Peace to thy manes, Brodum! if men allowed themselves to be duped by thy disciples, they richly deserved it. With you there was no disguise, no 'ignorance with looks profound,' no season or two under the lectures of Mr. A. or Dr. B. to quote, as a passport to the confidence of the new adventurer on your skill. All was straight-forward plain work. The sly general who physics the major part of the British community, if my whole speculation be not astray, as far out-herods the most professed quack, as hypocrisy, with an air of orthodoxy, is more dangerous than the broad cant of a jumping methodist." 135.

It is not our custom (as we observed in a former article) to flatter one class of medical society at the expense of another, but we must say, that our knowledge of medical practitioners, both in town and country, (and it is probably as extended as that of our more learned author,) by no means sanctions the correctness of the pencil which drew the foregoing portrait. If such characters exist, these delineations of them to the non-professional public will do no good, but a great deal of injury to the respectable practitioners, who form an overwhelming majority.

We dare not commit to our pages any more of these severe satires on the profession, because we think that the author will ultimately be sorry for having given them publicity in a work calculated for general perusal. The remedy which Sir Arthur proposes for all these maculæ in the fair face of human nature is, as we shewed a few pages back—a better education than medical practitioners now possess. To improvement of education, we would be among the last to object; but we know enough of human nature to be convinced, that no amount of *learning* will make men honest, where they have an inclination and a field for being otherwise—and this they will always have, both in law and in physic. Talents and education will, indeed, enable *such characters* to do the thing in a more masterly style, of which we have seen some notable instances among those who hold their heads high in physic. As long, in fact, as there are *fools* to be duped, there will be plenty of *knaves* to dupe them.

We must now pass on to the tenth chapter of the work, in which Sir Arthur discusses the charter, laws, &c. of the Royal College of Physicians, of which corporate body he is a FELLOW. Notwithstanding the influence of early partialities, not to say prejudices, our author "must candidly own that, for a long space, his eyes have not been shut to certain defects, which he has more than once hinted to the President his intention of some day submitting to the profession for the common benefit of all." This day, we suppose, is now arrived—and this exposition of defects, turns out to be almost entirely a laboured defence of the worst parts of the College laws and regulations, with some gentle chidings on certain foibles in the College, arising principally from the exuberance of its virtues and liberality!

*Answer to First Objection.* The *concealment* of the bye-laws, to the observance of which every fellow and licentiate is sworn on his bended knees, has been reprobated. To this the College advocate replies that, *as far as his recollection* serves him, no FELLOW is denied access to the bye-laws:—And as to the Licentiates, as they "are not associated in the government and business of the College, I cannot see it a hardship that *they* should not be freely allowed a liberty *which there is so much good reason to believe would often be vexatiously exercised.*" We believe the records of reasoning can hardly produce a parallel to the above piece of ratiocination! The Licentiates are sworn to obey laws which they are not allowed to see—and they are not allowed to see them, because there is good reason to believe that they would turn the knowledge thus obtained to purposes not quite palatable to the College! What must be the state of the College when one of its own FELLOWS puts forth such advocacy as this?

*Answer to Second Indictment.* The Fellows of the College are said to be in the habit of *consulting* with surgeons, apothecaries, &c. not authorised to be consulted with, according to the statutes.

"Now this I positively deny, without the least hesitation, *has ever been done with the sanction of the College;* or, if known, it has invariably been visited with the penalty awarded in similar cases of infringing the statute 'de conversatione morali.' That a FELLOW of the College may have been led, *under peculiar and pressing circumstances of a call upon his humanity, to enter into conference with persons of this description, I will not undertake to contradict.* In such a case, I should conceive he would be blamable in the extreme to refuse conferring even with a *common nurse.* This, however, is not *consultation.*" 288.

So, then, the Fellows of the College meet surgeons, or apothecaries, on the footing of common nurses, and that only on peculiar and pressing occasions! Is it possible that Sir Arthur can have been in his senses when he penned the above passage! Every man knows that the Fellows of the College enter daily into *long and familiar conference and consultation* with surgeons, and with apothecaries—nor does any sensible man blame them for so doing. In that point they shew their wisdom. But we blame them for consulting with these classes, and the next moment refusing to consult with a regular physician, who happens not to have paid the College 57 pounds for a license. This compliance on the one hand, and refusal on the other, cannot, we maintain, be attributed to any other than sordid, selfish motives, in despite of the following assertion.

"To suppose, as some have done, that such violations of duty are prompted by a sordid regard to self-interest, I conceive to be only the calumnious colouring of jealousy and ill-nature, and quite undeserving of notice." 289.

We leave the public to judge between the author and ourselves on this point.

*Answer to Third Indictment.* It is objected that the College does not now, as formerly, exercise its power in putting down irregular and empirical practitioners. This is admitted. But what is the excuse? Why, "that the persecution of heresy, instead of restraining, has served only to augment the number of offenders." How will Sir Arthur quadrate this principle with the prosecution of Dr. Harrison? The next objection we shall give in the words of the author, as the passage will shew that virtue itself, in excess, may become a fault.

"It is roundly asserted that the College have abandoned all concern about the country, not caring one farthing how it is eaten up by the rankest vermin of quackery; nay, that if they heard it was visited as severely as Pharaoh by his lice or locusts, they would not stir one step to stay the plague. *What to say to this, I own myself rather at a stand.* That they possess the same right to repress the evil in both town and country, *I have the best authority for stating to be fact;* and it surely cannot be their opinion that the health of his Majesty's country subjects is not on a par of importance to the state with the health of his good city and suburbs of London, to the seventh mile stone. I freely admit that I see something here not quite as it should be; and for consistency sake, if there were no better motive, the College are, in my judgment, very positively called upon to account for this their *greater tenderness* to the town." 291.

Notwithstanding the excellence of Sir Arthur's authority for the power of the College over the country as well as the town, we can give him a little information which will let in a flood of light on his mental optics respecting the "greater tenderness" to the town. The College has no power beyond the seventh mile stone—and for this simple reason, that there is *no penalty* annexed to practising without its permission there; and without a penalty, a law is not of much use. Does Sir Arthur now see why the "lice and locusts" of the country are spared? We can assure him that the College would pounce upon the Harrisons of Cheltenham as well as upon those of London, if they had the smallest power! The reason why they spare the "lice and locusts" of London—or, in other words, the illegitimate practitioners or quacks, is, that they would probably be out of pocket by prosecuting them—and, after all, could not compel them to pay the 57 pounds. Hence it is that their power is aimed at those who attempt the higher walks of the profession without first paying the tax that so materially keeps up the Temple in Pall-Mall East, and at the same time maintains the distinction between them and their equals, the Licentiates.

*Last Objection answered.* Sir Arthur observes that the "detractors" of the College object to Oxford and Cambridge, as schools of physic, because medical science is not taught there. In answer to this he says—"They do not profess—they were, in fact, not established—to qualify a man fully for the practice of physic." They were designed for giving a ground-work in literature and science. Very well. We shall take Sir Arthur on his own ground. A few pages farther on he maintains that the *medical* degree obtained at Oxford or Cambridge is a perfect test of medical education, and therefore the graduates of these universities *ought not* to undergo any examination by the College of Physicians before they practise in London! Was there ever such inconsistency! "And truly, if the degrees of these universities cannot be relied upon as vouchers, their endorsement by the College will scarcely deserve to be regarded as much better security. Without all question it appears as if something here required the hand of correction." Thus, then, a diploma from a university where no medical education is given may be relied upon as a voucher, without any examination; but a diploma from Edinburgh or Paris, where the strictest discipline and the most rigid examination are enforced, goes for nothing! Sir Arthur rails in good set terms against this ordeal of the College imposed on the men of Oxford and Cambridge; though, in our humble opinion, it is one of the best statutes in the whole of their laws. We maintain that the tests should be two-fold—proofs of study, both general and medical, in some proper university—and a rigid examination to ascertain whether those studies had been turned to advantage.

Sir Arthur next goes into a long discussion on the merits of the Edinburgh school, grounding his objections to that school on a long letter to the patrons of the university, by some Edinburgh doctor, whose name is not given. In singling out this witness, Sir Arthur emphatically observes—"I like to shew every fair play." Now to our apprehension, the way to shew fair play is to take the sentiments of a stranger—of an unbiassed spectator, and not the declamation of a professed partisan. Sir Arthur appears to be like the Turkish Cadi—he never wishes to hear more than one side of a question. The "*audi alteram partem*" would only tend to puzzle and perplex! He takes not the least notice of the able answer which the younger Duncan has given to the letter of—we believe, Dr. Thomson. Not but that we coincide in the sentiments contained in this memorial respecting a better education for the medical practitioner. We believe that all regulations will be defective where a good classical and general education is not made a *sine qua non*. But the doctrine of limiting the acquisition of this education to two or three universities is preposterous, and must soon be scouted by the public at large.

But to return to the College. Sir Arthur, after paying a just compliment to the President, laments that the interior economy of this stately pile by no means comports with its exterior magnificence.

"Now three years have elapsed, and things are just as they were, with the unhappy difference, that placed more prominently, it has been made to excite more envy, and a narrower inspection, and a closer dissection of its defects. But in truth, the error is not so much in the College as in our Universities. It is there the main reformation ought to commence; and the only reformation required, is simply to raise the respectability of their medical degrees. I should say, let no degree be henceforth conferred by any University in the kingdom, that is not fully answerable to what it sets forth regarding the bearer, in every respect, both of character and competency. The only authority the College of Physicians would then be required to use, in reference to the graduates of any University, would be that of merely verifying the authenticity of their degrees, and of enrolling them in a list to be published regularly for the information of the public.

"In raising the respectability of the University degrees, it would be necessary to have it surely settled that the candidate should be able to produce certificates of his having passed through some established course of medical instruction, under some competent authority, that the Universities could recognize whether within the walls of an University or elsewhere. This measure would furnish a supply of competent medical practitioners to meet the demand of the public, and effectually rescue the profession of the physician from the hands of its spurious pretenders. *No one province of the profession would then trench upon another.*" 319.

This is so liberal, that we began to wonder how we could have so much mistaken the aim and end of this publication. But we were soon undeceived. In the very next page we find the Corporation spark kindle into a bright flame.

"As to the constitution of the College itself, I conceive it most important and essential that it (the Fellows of course) should be composed of men of *a degree of learning and acquirement much above that wanted*

*for the fitness of the ordinary members* (Licentiates) of the profession. It is only by keeping up a superior rank of this description that the profession can be saved from the same degeneracy that has been so universally its portion in all other parts of the world, and to which it has itself so natural and prone a tendency." 320.

So, then, by keeping a handful of men educated at particular seats of learning, in possession of rank, power, and privileges, to domineer over all others, is the way to prevent degeneracy—to promote harmony—to ensure liberality—and to counteract that tendency to descend—"especially if tempted and drawn by the divellent attraction of *self-interest.*" Now we do maintain that such a distinction, founded as it is, not upon superior learning, talent, medical acquirements, or moral worth, but upon certain corporation rights attached to insulated seats of learning, is the very apple of discord which will keep up a constant source of arrogance, pride, and tyranny in one party; and just indignation, if not deadly hatred, in the other, as long as such an odious barrier exists.

Sir Arthur is an excellent classical scholar, and seems to be an enthusiastic admirer of the lions of literature in ancient times. We ask him if it was by means of corporation laws that his favourites of the olden time, the Platos, Aristotles, Horaces, and Virgils of Greece and Italy, were enabled to "keep up a superior rank" above their cotemporaries? Was it by virtue of this corporation supremacy that *Licentiate* Sydenham has transmitted his name to posterity, in every language of the world, while the Radcliffes, and other FELLOWS of his time, have only a momentary and despised existence in the Mems. Maxims, and Memoirs of Wadd? If, indeed, the fellowship of the College were a station of eminence to which talent, learning, and professional acquirements had a right to aspire, as a reward, and to which nothing else could give a claim, there might be some foundation for the observation of our author. But he and every body knows that merit has no claim to the fellowship, and that the paths to it are two particular *highways*, lying between the metropolis and two provincial towns! Is this the *way* to forward merit and foster talent in the nineteenth century?

At page 155, Sir Arthur tells us, that—

"If the College of Physicians had never been of any use beyond that of keeping up a reputable rank in the profession, it is worthy of all its power and patronage."

Yet, at page 321, we have the following somewhat contradictory declaration.

"As things have for a long time been conducted, the authority of the College in the country is absolutely a dead letter, and its testimonial, in place of a benefit, a positive nuisance; in fact, it is a millstone about the neck of the bearer, by imposing obligations at variance with his private interests, and so far putting bad faith at a premium."

How the author can reconcile the above passages, it would be difficult to imagine; nor do we well understand how the fellowship hangs as a millstone round the neck of a physician in the country. But this we may surely say, that the "testimonial" of the College hangs as a millstone about the neck of the Licentiate in London.

In thus offering a critique on the *medical politics* of the volume before us, we beg it may be distinctly understood, that we mix up no personal feelings as regards the author. We have every reason to believe, and, indeed, to know, that Sir Arthur Faulkner is an accomplished scholar, a talented physician, and an honorable man. His work on the Plague has long made his name favourably known to the profession, and the present volume proves him a man of literature and observation. But these good qualities cannot always clear the mental optics, when things are to be viewed through the medium of medico-political lights and shades. Every man is entitled to his opinion on these topics. We may be wrong, and our author may be right. The public tribunal is that to which we shall appeal, firmly, but we hope freely; alike uninfluenced by the smiles of one party, or the frowns of the other.

